# Masquerading Bundle Branch Block: A Critical ECG Pattern Hidden in Plain Sight

**DOI:** 10.7759/cureus.97262

**Published:** 2025-11-19

**Authors:** Selma Siagh, Meriem Bouali, Malak Amrani, Jamal Kheyi, Hicham Bouzelmat

**Affiliations:** 1 Department of Cardiac Electrophysiology, Mohammed V Military Teaching Hospital, Mohammed V University, Rabat, MAR

**Keywords:** atrioventricular block, bifascicular block, conduction system disease, left bundle branch block, masquerading bundle branch block, right bundle branch block

## Abstract

Masquerading bundle branch block (MBBB) is a rare and often overlooked electrocardiographic (ECG) pattern characterized by right bundle branch block (RBBB) morphology in the right precordial leads combined with features resembling left bundle branch block in the limb leads and/or the left precordial leads. This pattern reflects bilateral conduction system disease and carries a significant risk of progression to high-grade atrioventricular block (AVB). We report the case of an 84-year-old woman admitted for acute decompensated heart failure with preserved ejection fraction and atrial flutter. Her ECG showed wide QRS complexes with RBBB features in the precordial leads, including an rsR’ pattern in leads V1-V2 and slurred S waves in leads V5-V6, along with absent S waves in leads I and aVL, consistent with the standard type of MBBB. Transthoracic echocardiography revealed concentric left ventricular hypertrophy with preserved systolic function and elevated filling pressures. After successful cardioversion of the atrial flutter, the electrophysiological study confirmed trifascicular block with a prolonged His-ventricular (HV) interval at 68 ms. Given the absence of clinical symptoms and an HV interval remaining below the 70-ms threshold, the patient was deemed suitable for discharge. However, in light of the known risk of progression to high-grade AVB in patients with MBBB, a close follow-up was scheduled, including planned reassessment and consideration for pacemaker implantation if indicated. This case underscores the importance of recognizing MBBB, a pattern that is frequently overlooked despite its potential prognostic value. It remains insufficiently addressed in current guidelines. Enhancing awareness of this underrecognized entity is essential to ensure timely diagnosis, appropriate risk stratification, and optimal management.

## Introduction

Masquerading bundle branch block (MBBB) is an electrocardiographic (ECG) pattern characterized by right bundle branch block (RBBB) morphology in the right precordial leads, combined with features suggestive of left bundle branch block (LBBB) in the limb leads and/or the left precordial leads [1-3]. It is a rare finding, reported in 4-16 ECGs per 100,000 [4,5]. MBBB is typically associated with extensive conduction system disease and carries a high risk of progression to high-grade atrioventricular block (AVB) [4-6]. Despite its clinical relevance, it is often underdiagnosed due to limited awareness among clinicians, which may result in suboptimal management. It may also contribute to the likely underestimation of its true prevalence. Moreover, in contrast to other forms of plurifascicular conduction system disease, MBBB is not specifically addressed in the 2021 European Society of Cardiology (ESC) Guidelines on Cardiac Pacing and Cardiac Resynchronization Therapy [7]. A thorough recognition and understanding of this ECG pattern is therefore essential, as it may have direct implications for risk stratification and early consideration for permanent pacemaker implantation in appropriate patients. Here, we present the case of an 84-year-old patient who was admitted for acute decompensated heart failure with preserved ejection fraction (HFpEF) and atrial flutter. The ECG revealed an MBBB pattern in an otherwise asymptomatic patient. Following electrophysiological testing, no pacing was indicated. However, a close follow-up was arranged, given the potential risk of progression to high-grade AVB.

## Case presentation

An 84-year-old woman with a history of hypertension treated with ramipril (5 mg/day) was admitted to the cardiology department for shortness of breath. Over the past several months, she had experienced progressively worsening shortness of breath, which had significantly limited her daily activities in recent days. She also reported multiple episodes of palpitations over the past year, lasting several minutes to hours and occurring both at rest and during exertion.

On admission, the patient was fully conscious with a Glasgow Coma Scale score of 15. She was hemodynamically stable, with a blood pressure of 118/49 mmHg and a heart rate of 98 beats/minute. Notably, the patient was orthopneic and tachypneic, with an oxygen saturation of 89% on room air. Pulmonary auscultation revealed bilateral crackles. There were no signs of right heart failure, and cardiac auscultation was unremarkable. Finally, both calves and thighs were soft and non-tender.

**Figure 1:**
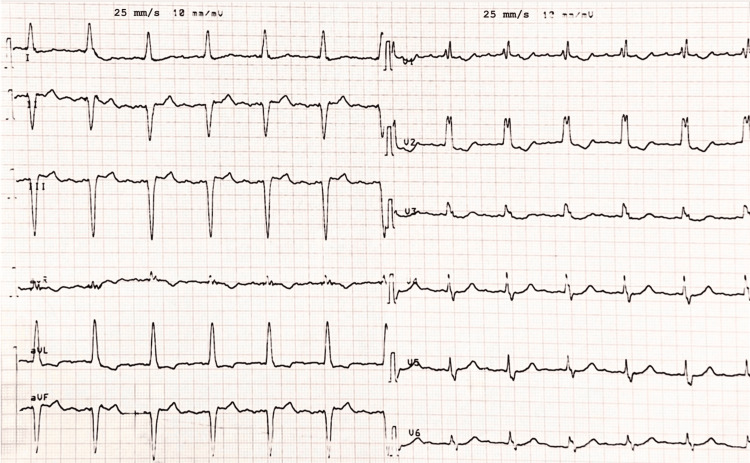
Twelve-lead ECG showing left atrial flutter with 4:1 conduction and wide QRS complexes with features consistent with masquerading bundle branch block. The ECG shows an atrial rhythm at 300 beats/minute with 4:1 conduction, low-amplitude flutter waves in the inferior leads, and positive waves in V1, findings suggestive of either typical counterclockwise atrial flutter or left atrial flutter (with the latter subsequently confirmed during electrophysiological study), while a focal atrial tachycardia could not be excluded. QRS complexes are wide (120 ms), with a right bundle branch block morphology in the precordial leads (rsR′ pattern in lead V1 and broad S waves in leads V5-V6). The limb leads demonstrate absence of S waves in leads I and aVL, and loss of septal Q waves in lead I, features consistent with a left bundle branch block-like pattern. Left axis deviation is noted, along with an rS pattern in the inferior leads.

The ECG on admission (Figure 1) showed an atrial rhythm at approximately 300 beats/minute with 4:1 conduction, resulting in an average ventricular rate of 75 beats/minute. Low-amplitude flutter waves were noted in the inferior leads, but their polarity was difficult to assess because of the low voltage. In contrast, positive atrial deflections were clearly observed in lead V1. These findings were suggestive of either typical counterclockwise atrial flutter or left atrial flutter, although focal atrial tachycardia could not be excluded on surface ECG. The diagnosis of left macroreentrant atrial tachycardia was subsequently confirmed during the electrophysiological study. Importantly, the QRS complexes were wide, measuring 120 ms. The precordial leads displayed features consistent with RBBB, including an rsR′ pattern in lead V1 and slurred S waves in leads V5 and V6. Notably, in the limb leads I and aVL, the typical slurred S waves associated with RBBB were absent. Instead, these leads exhibited features consistent with LBBB, such as broad monomorphic R waves, along with the absence of septal Q waves in lead I. A left axis deviation was also noted, with an rS pattern in the inferior leads. Taken together, these findings fulfilled the ECG criteria for MBBB. Finally, voltage criteria for left ventricular hypertrophy were met with an R wave in aVL exceeding 11 mm.

Chest X-ray revealed bilateral hilar congestion, suggestive of acute pulmonary edema. Transthoracic echocardiography showed concentric left ventricular hypertrophy and a slightly dilated left atrium measuring 21 cm², with preserved left and right ventricular systolic function and no significant valvular abnormalities. Filling pressures were elevated. Finally, laboratory tests were within normal limits (Table 1).

**Table 1:** Initial laboratory findings. Electrolytes and thyroid-stimulating hormone were within normal limits, ruling out reversible metabolic or endocrine causes of conduction abnormalities. Additionally, complete blood count, renal function, and transaminases provided baseline data before anticoagulant initiation.

Laboratory test	Result	Reference value
Hemoglobin (g/dL)	12.2	12.0–16.5
White blood cells (×10^9^/L)	6.0	4.0–10.0
Platelets (×10^9^/L)	199	150–400
Sodium (mmol/L)	140	135–145
Potassium (mmol/L)	3.9	3.5–5.0
Chlorine (mmol/L)	98	98–107
Bicarbonates (mmol/L)	22	22–26
Calcium (mg/dL)	9.2	8.0–10.0
Magnesium (mg/dL)	2.4	1.6–2.5
Urea (mg/dL)	37	15–55
Creatinine (mg/dL)	1.1	0.7–1.3
Aspartate transaminase (IU/L)	15	5–34
Alanine transaminase (IU/L)	12	0–55
Thyroid-stimulating hormone (mIU/L)	1.2	0.5–5.0

The patient underwent an invasive electrophysiological study that identified a regular atrial rhythm with a cycle length of 200 ms (corresponding to an atrial rate of approximately 300 beats/minute). Intracardiac recordings were obtained using a decapolar catheter positioned in the coronary sinus, with proximal electrodes bracketing the coronary sinus ostium, and a duodecapolar Halo catheter positioned along the tricuspid annulus. The activation sequence in the coronary sinus was distal-to-proximal, strongly suggesting a left atrial origin of the arrhythmia. Cycle length variability was less than 2% on a beat-to-beat basis, and atrial activation spanned the entire tachycardia cycle length (TCL), findings consistent with a macroreentrant mechanism rather than a focal tachycardia. Overdrive pacing was performed, and entrainment of the tachycardia was achieved. Entrainment from the cavotricuspid isthmus produced a long post-pacing interval (PPI), with a PPI-TCL difference of about 150 ms, indicating that this structure was not part of the reentrant circuit. In contrast, entrainment from the distal coronary sinus resulted in a small PPI-TCL difference of approximately 16 ms, indicating proximity to the reentrant circuit and confirming a left atrial macroreentrant atrial tachycardia. As three-dimensional electroanatomical mapping systems are not available in our laboratory, the diagnosis of left atrial flutter was established using conventional intracardiac recordings, coronary sinus activation sequence, and entrainment maneuvers, which remain validated methods for the identification of macroreentrant atrial circuits. Given that this was the first documented episode of symptomatic left atrial flutter and our center does not routinely perform ablation of left atrial flutters, the patient underwent electrical cardioversion.

Following restoration of sinus rhythm, an evaluation of the conduction system was performed. Intracardiac recordings were obtained using standard diagnostic catheters positioned at the His bundle region, right atrium, and right ventricular apex. Baseline measurements showed an atrial-His (AH) interval of 120 ms, at the upper limit of the normal range (NR = 50-120 ms), and a prolonged His-ventricular (HV) interval of 68 ms (NR = 35-55 ms), consistent with significant infra-Hisian conduction delay. Taken together, the surface ECG and intracardiac findings were consistent with trifascicular block, characterized by complete RBBB and left anterior fascicular block (LAFB), associated with a prolonged HV interval. As the patient remained asymptomatic, with no history of syncope or presyncope, and the HV interval was below the 70-ms threshold, pacemaker implantation was not performed.

**Figure 2:**
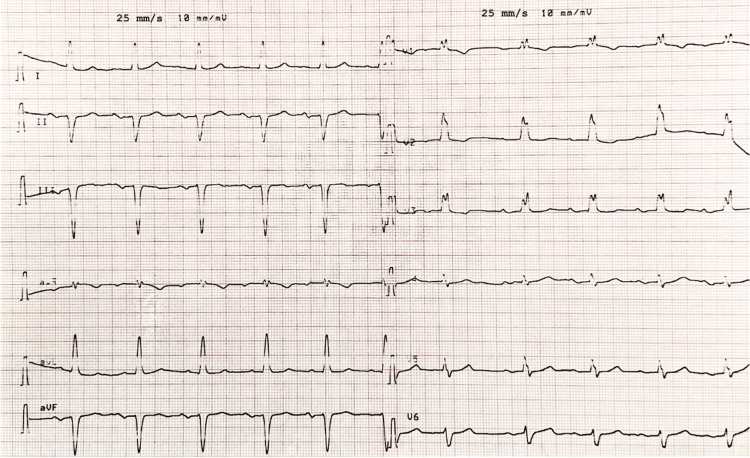
Post-procedural ECG after cardioversion of left atrial flutter showing sinus rhythm with advanced interatrial block, first-degree atrioventricular block, and masquerading bundle branch block. The ECG shows a regular sinus rhythm at 67 beats/minute with advanced interatrial block (P-wave duration of 150 ms and biphasic morphology in leads II, III, and aVF). A first-degree atrioventricular block is present with a prolonged PR interval of 240 ms. QRS complexes are wide (120 ms) with left axis deviation and a masquerading bundle branch block pattern, unchanged compared with the admission ECG.

The post-procedural ECG obtained after cardioversion of atrial flutter (Figure 2) demonstrated a regular sinus rhythm at 67 beats/minute. The P-wave duration was prolonged at 150 ms (NR = <120 ms), with a biphasic (positive-negative) morphology in the inferior leads (II, III, aVF), findings consistent with advanced interatrial block (A-IAB). First-degree AVB was also present, with a prolonged PR interval of 240 ms. The QRS morphology remained unchanged compared with the admission ECG, showing wide QRS complexes (120 ms), left axis deviation, and an MBBB pattern, thereby excluding conduction aberrancy related to atrial flutter.

The patient was discharged on apixaban 5 mg twice daily, based on a CHA₂DS₂-VA score of 4, along with empagliflozin and her ongoing antihypertensive medication. Ambulatory ECG monitoring over 72 hours documented paroxysmal atrial fibrillation (AF), but no episodes of paroxysmal AV block were recorded. A strategy of close clinical follow-up was adopted, with consideration for potential future implantation of a dual-chamber pacemaker if necessary.

## Discussion

MBBB is a term first introduced by Richman and Wolff in their 1954 publication in the American Heart Journal, in which they described four cases exhibiting a distinctive electrocardiographic pattern: LBBB morphology in the limb leads coexisting with RBBB features in the precordial leads [1]. This is currently referred to as the standard type of MBBB. A second variant, known as the precordial type, is also now recognized and is defined by RBBB morphology in the right precordial leads with concurrent LBBB features in the left precordial leads [2,3]. MBBB is a relatively rare pattern. In fact, Bayés de Luna et al. identified only 16 cases after reviewing 100,000 ECGs [4], while Schroder e Souza et al. found 25 cases among 600,000 ECGs [5], indicating a prevalence ranging from 0.04 to 0.16 per 1,000. However, its true prevalence may be underestimated due to the widespread under-recognition of this ECG pattern, as it remains largely unfamiliar to clinicians.

To establish a more precise definition of this entity, we reviewed the diagnostic criteria reported in the literature. For the diagnosis of the standard type of MBBB, which is the most frequently described and corresponds to the subtype observed in our case, the features most consistently cited include wide QRS complexes (≥120 ms), a typical RBBB pattern in the precordial leads (including rsr′, rsR′, or rSR′ patterns or, less commonly, a wide and notched R wave in leads V1-V2 along with slurred S waves ≥40 ms in leads V5-V6), as well as absent or minimal S waves (<1 mm) in the limb leads) [1-3]. Moreover, left axis deviation with LAFB morphology is commonly reported [2-4]. These criteria have since been adopted as inclusion criteria in several studies [4-6,8]. Finally, regarding septal Q waves in the lateral limb leads, Rosenbaum et al. specifically emphasized the absence of a Q wave in lead I, consistent with the classical definition of LBBB [2]. However, in the original description by Richman and Wolff [1], as well as in a later publication by Elizari et al. [3], the presence of a small Q wave in leads I and aVL was considered possible. While most studies do not explicitly comment on septal Q waves, they are generally not observed on visual analysis of the ECG tracings [4-6,8]. In our case, the diagnosis of the standard type of MBBB was established based on ECG findings that fulfilled all previously described criteria, including a QRS duration of 120 ms, an RBBB pattern in the precordial leads, absence of S waves in the frontal leads I and aVL, and absence of septal Q waves in lead I.

**Figure 3:**
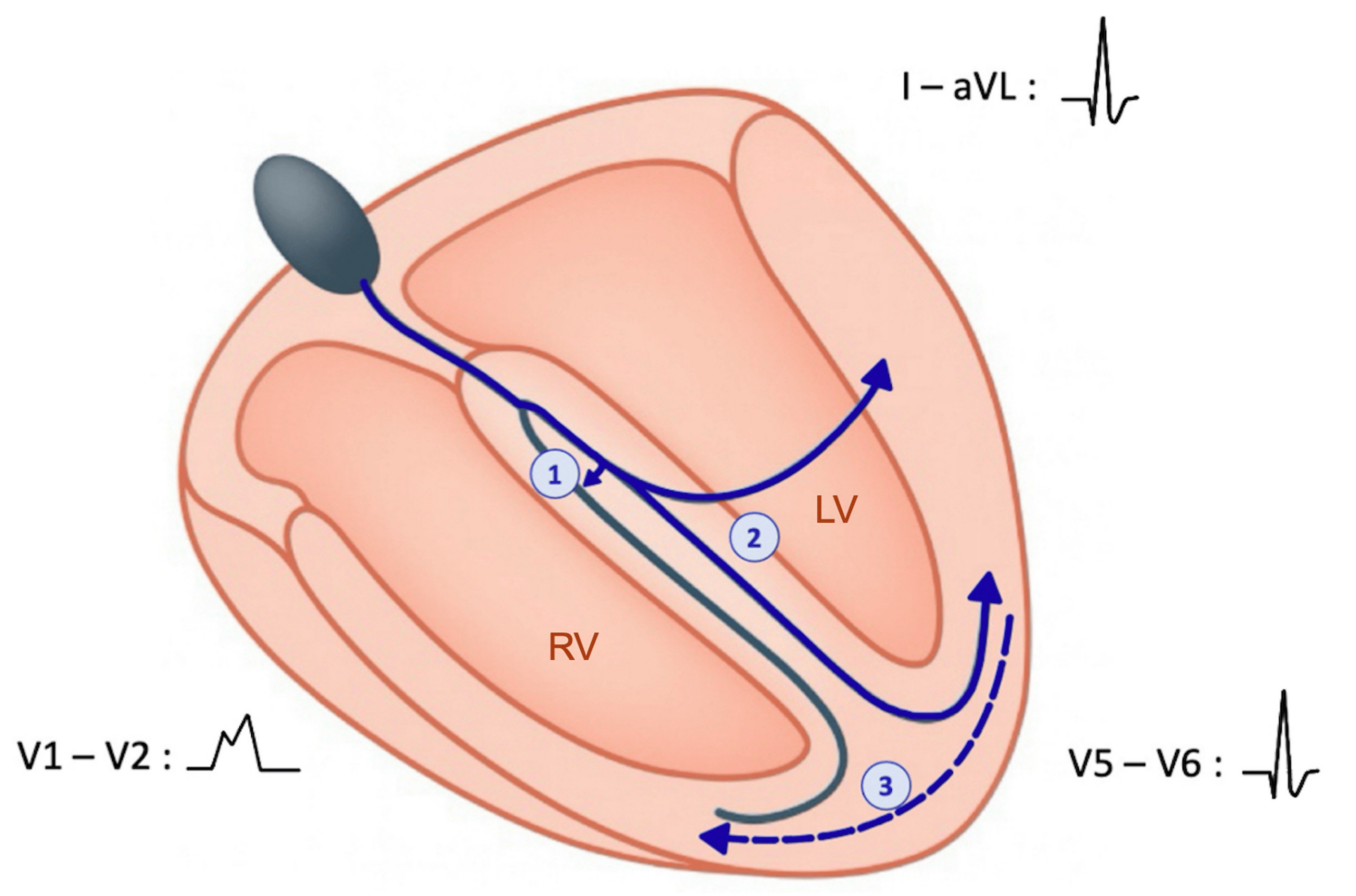
Schematic cross-sectional view of the heart illustrating ventricular depolarization in right bundle branch block. Original illustration by the authors. The arrows labeled “1” and “2” illustrate the propagation of the electrical impulse through the His-Purkinje system, with the numbers indicating the sequence of depolarization: first septal depolarization, and then left ventricular activation, both via the left bundle branch. The dashed arrow labeled “3” represents the slow right ventricular depolarization via cell-to-cell conduction, which accounts for the slurred S waves observed in the lateral leads. The electrocardiographic leads (V1-V2, V5-V6, I-aVL) are positioned relative to the myocardial regions they explore and display the corresponding QRS complexes. LV: left ventricle; RV: right ventricle

**Figure 4:**
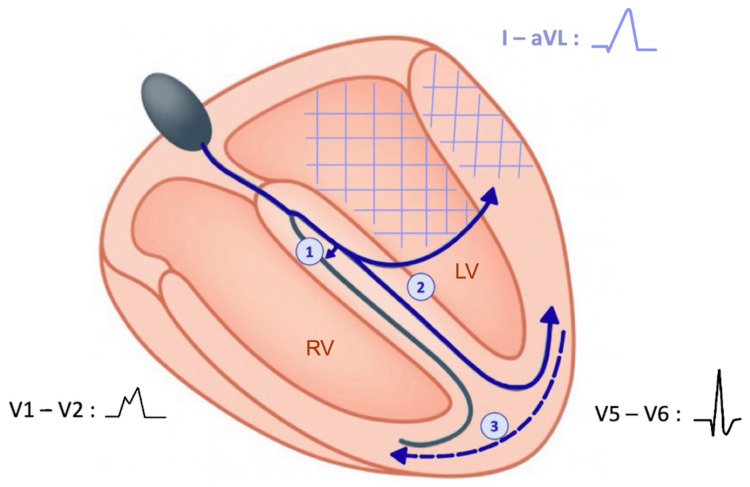
Schematic cross-sectional view of the heart illustrating ventricular depolarization in the standard type of masquerading bundle branch block. Original illustration by the authors. The cross-hatched area indicates a region of delayed activation in the anterolateral wall of the left ventricle. This delay overlaps with the late right ventricular activation (dashed arrow) rendering it no longer unopposed and thereby masking the terminal S-wave component in leads I and aVL. Conventions are consistent with those used in Figure 3.

To better understand the prognostic significance of this ECG pattern, we examined its electrophysiological basis, as it provides insight into the extent of conduction system disease. The standard type of MBBB results from a conduction delay or block in the right bundle branch, producing a typical RBBB morphology in the right precordial leads. This is accompanied by delayed activation of the left ventricle, particularly in its anterolateral region. As a consequence, late right ventricular activation is no longer unopposed in this area, leading to the attenuation or disappearance of the characteristic slurred S wave in leads I and aVL [3,8,9]. Additionally, the terminal electrical forces of the QRS complex become reoriented leftward and superiorly, which explains the left axis deviation frequently associated with this pattern [3] (Figures 3, 4).

The key unresolved issue remains the underlying cause of delayed left ventricular activation in MBBB. Since its initial description in the 1950s, the pathophysiological basis of this pattern has been widely debated, yet no definitive explanation has been established. Nevertheless, several plausible hypotheses have emerged in the literature. A landmark article published in Circulation in 1958 by Unger et al. laid the groundwork for many subsequent theories. Based on autopsy findings from two patients who exhibited this ECG pattern, the authors identified three main abnormalities: incomplete but significant impairment of both the right and left bundle branches (the most prominent finding), myocardial fibrosis or necrotic scarring affecting the septum and, to a lesser extent, the left ventricular free wall, and the presence of left ventricular hypertrophy [10]. These mechanisms have since been consistently cited as potential contributors to the development of the MBBB pattern.

Currently, the prevailing hypothesis posits incomplete conduction impairment affecting both the right and left bundle branches, resulting in bilateral intraventricular conduction delay [2,9,10]. Herweg et al. described this phenomenon as bilateral first-degree bundle branch block [9], while Rosenbaum et al. referred to it more broadly as bilateral bundle block [2]. Alternatively, an MBBB pattern may result from a complete block of the right bundle branch combined with conduction delay in the left bundle branch [2,11]. These hypotheses were initially based on correlations between ECG findings and postmortem histopathological observations [2,9,10]. More recently, electrophysiological studies have further supported these mechanisms. Notably, Tzogias et al. examined 2,253 patients who developed a new RBBB pattern in the right precordial leads following unintentional catheter-induced trauma to the right bundle branch. Patients were categorized based on their baseline ECGs into three groups: normal, fascicular block (including LAFB and left posterior fascicular block), and LBBB. The ECG changes following RBBB onset were then analyzed. A key finding was that the absence of S waves in leads I and aVL during RBBB was 100% specific for pre-existing LBBB. The authors concluded that this pattern likely results from either a conduction delay in the left bundle branch combined with complete RBBB, or bilateral conduction delays of varying degrees [11]. Similar findings were reported by Herweg et al., who observed comparable ECG changes after traumatic RBBB induction, further supporting the concept of bilateral conduction system disease in MBBB [9]. Any component of the left bundle branch may be involved. The left anterior fascicle is most frequently affected, which may explain the commonly observed left axis deviation [3,8]. Pre-divisional involvement of the left bundle branch has also been described and may account for the absence of septal Q waves in lead I, as it alters the early phase of ventricular activation [8,9]. Additional mechanisms that may contribute to delayed anterolateral left ventricular activation include left ventricular hypertrophy and focal conduction block, as previously proposed by Unger et al. [10]. Such a focal block may result from areas of infarction or fibrosis, commonly observed in conditions such as cardiomyopathies and Chagas disease [3,10]. Finally, regarding the less common precordial variant of MBBB, its pathophysiological basis remains incompletely defined. Similar mechanisms have been proposed, including marked LAFB, with or without coexisting left ventricular hypertrophy, as well as anterior wall conduction delay due to prior infarction or fibrosis [3].

In our case, several factors may account for the observed standard-type MBBB pattern. Bilateral conduction system disease is consistent with the ECG findings of broad QRS complexes showing RBBB and LAFB morphology. Trifascicular block was confirmed during the electrophysiological study by a prolonged HV interval. Furthermore, a possible pre-divisional involvement of the left bundle branch, supported by the absence of septal Q waves in lead I, can be another contributing factor. The LAFB likely played a pivotal role in the delayed activation of the anterolateral region of the left ventricle, whereas coexisting left ventricular hypertrophy may also have contributed to the MBBB pattern. Of note, pressure overload-related left ventricular hypertrophy may additionally explain the loss of septal Q waves in the lateral leads.

The key takeaway is that MBBB is most frequently associated with conduction system impairment involving both bundle branches, a finding that logically correlates with an increased risk of AVB. To further explore this issue, we conducted a focused review of the available epidemiological studies on this topic. We identified and summarized the key clinical and epidemiological characteristics of patients presenting with this specific ECG pattern (Table 2). The core findings include a clear male predominance and an average age typically over 70 years [4-6]. Additionally, the most common underlying conditions are ischemic heart disease [4-6] and hypertension [4]. However, a notable proportion of patients, 9% according to Gómez Barrado et al. [6] and 12% according to Bayés de Luna et al. [4], have isolated conduction system disease.

**Table 2:** Reported characteristics of patients with masquerading bundle branch block in published epidemiological studies since 1988. F: female; M: male; NS: not specified

Studies	Bayés de Luna et al. (1988) [4]	Gómez Barrado et al. (1997) [6]	Schroder e Souza et al. (2015) [5]
Sex ratio (M:F)	4:1	10:1	5:1
Average age (in years)	70 ± 9	73 ± 10	69 ± 14
Ischemic heart disease	68%	59%	NS
Dilated cardiomyopathy	13%	NS	NS
Hypertensive heart disease	6%	55%	NS
Aortic stenosis	NS	9%	NS
No structural heart disease	13%	9%	NS

Of note, our patient exhibited both atrial flutter and AF, prompting us to investigate the potential association between MBBB and atrial arrhythmias. Gómez Barrado et al. reported that 59% of patients in their cohort had an associated atrial arrhythmia, either chronic or paroxysmal, including AF, atrial flutter, or atrial tachycardia [6]. Similarly, in the study by Schroder e Souza et al., atrial arrhythmias were observed in 32% of patients, with AF accounting for 28% and atrial flutter for 4% of cases [5]. This association may be explained by shared underlying factors, as both MBBB and atrial arrhythmias are commonly encountered in older patients and are often linked to left ventricular disease, such as coronary artery disease, hypertension, or heart failure [5,6]. Notably, an important finding was the presence of A-IAB on the patient’s sinus rhythm ECG, although this was beyond the primary focus of this report. The association between A-IAB and atrial arrhythmias, particularly AF but also atrial flutter and atrial tachycardia, has been described as Bayés syndrome. This is consistent with the fact that both atrial arrhythmias and A-IAB share atrial fibrosis as a common substrate. This condition has been associated with an increased risk of stroke, dementia, and mortality [12].

Finally, regarding the long-term prognosis of patients with MBBB, follow-up data from the aforementioned studies are summarized in Table 3. The progression to advanced AVB was observed in over 50% of cases, with reported pacemaker implantation rates ranging from 41% to 68% [4-6]. This contrasts markedly with the annual progression rate of only 1% to 4% typically reported in patients with classical bifascicular block [13]. Furthermore, in the study by Bayés de Luna et al., the mean time from the diagnosis of MBBB to pacemaker implantation was 11.3 ± 22 months [4].

**Table 3:** Long-term outcomes of patients with masquerading bundle branch block based on follow-up data from published epidemiological studies since 1988. AVB: atrioventricular block; NS: not specified

Studies	Bayés de Luna et al. (1988) [4]	Gómez Barrado et al. (1997) [6]	Schroder e Souza et al. (2015) [5]
Follow-up period (in months)	72	32	48
High-degree AVB	56%	59%	NS
Pacemaker implantation	62%	68%	41%
Death (all cause)	43%	26%	39%

Our case is noteworthy as it draws attention to an often underrecognized ECG pattern: MBBB. This pattern suggests the presence of advanced bilateral conduction system disease and carries important diagnostic, prognostic, and therapeutic implications. In the present case, the patient was asymptomatic, and an electrophysiological study revealed trifascicular block with an HV interval of 68 ms (below the 70-ms threshold). In the absence of specific recommendations for MBBB in the current ESC guidelines, no pacemaker was implanted. This decision was consistent with the guidelines section on pacing in bundle branch blocks [7]. However, awareness of this ECG pattern and its high risk of progression to high-grade AVB warrants close clinical monitoring and may justify early consideration for pacemaker implantation. Therefore, regular clinical and ECG follow-up was arranged, and clear patient and family education was provided to seek prompt medical evaluation in the event of syncope or presyncope. This strategy aims to ensure timely pacemaker implantation if indicated. Nonetheless, further clinical experience is required to better define the full spectrum and natural history of MBBB and to inform evidence-based management strategies.

## Conclusions

MBBB is an uncommon and often underrecognized ECG pattern that reflects advanced bilateral conduction system disease. Among its variants, the standard type, as observed in our case, is the most frequently reported. This pattern typically results from the coexistence of RBBB and marked conduction delay within the left bundle branch. This leads to delayed activation of the anterolateral region of the left ventricle, thereby masking the slurred S wave in the high lateral leads. MBBB is associated with a high risk of progression to high-grade AVB, often requiring early consideration for pacemaker implantation. Although considered rare in the literature, its true prevalence is likely underestimated due to limited clinician awareness. Moreover, its management remains insufficiently defined in clinical practice despite its significant prognostic implications. By reporting this case, we aim to increase awareness of this distinctive ECG entity and highlight the importance of early recognition and appropriate risk stratification. Ultimately, further studies are warranted to better define the natural history of MBBB, assess its prognostic value, and establish clear management strategies.
